# Significance of Inactivated Genes in Leukemia: Pathogenesis and Prognosis

**DOI:** 10.22074/cellj.2017.4908

**Published:** 2017-05-17

**Authors:** Nazanin Heidari, Saeid Abroun, Jessika Bertacchini, Tina Vosoughi, Fakher Rahim, Najmaldin Saki

**Affiliations:** 1 Health Research Institute, Thalassemia and Hemoglobinopathy Research Center, Ahvaz Jundishapur University of Medical Sciences, Ahvaz, Iran; 2 Department of Hematology, Faculty of Medical Sciences, Tarbiat Modares University, Tehran, Iran; 3 Signal Transduction Unit, Department of Surgery, Medicine, Dentistry and Morphology, University of Modena and Reggio Emilia, Modena, Italy

**Keywords:** Leukemia, Gene Silencing, Tumor Suppressor, Pathogenesis, Prognosis

## Abstract

Epigenetic and genetic alterations are two mechanisms participating in leukemia, which can inactivate genes involved in leukemia pathogenesis or progression. The purpose of this review was to introduce various inactivated genes and evaluate their possible role in leukemia pathogenesis and prognosis. By searching the mesh words “Gene, Silencing AND Leukemia” in PubMed website, relevant English articles dealt with human subjects as of 2000 were included in this study. Gene inactivation in leukemia is largely mediated by promoter’s hypermethylation of gene involving in cellular functions such as cell cycle, apoptosis, and gene transcription. Inactivated genes, such as *ASPP1, TP53, IKZF1* and *P15*, may correlate with poor prognosis in acute lymphoid leukemia (ALL), chronic lymphoid leukemia (CLL), chronic myelogenous leukemia (CML) and acute myeloid leukemia (AML), respectively. Gene inactivation may play a considerable role in leukemia pathogenesis and prognosis, which can be considered as complementary diagnostic tests to differentiate different leukemia types, determine leukemia prognosis, and also detect response to therapy. In general, this review showed some genes inactivated only in leukemia (with differences between B-ALL, T-ALL, CLL, AML and CML). These differences could be of interest as an additional tool to better categorize leukemia types. Furthermore; based on inactivated genes, a diverse classification of Leukemias could represent a powerful method to address a targeted therapy of the patients, in order to minimize side effects of conventional therapies and to enhance new drug strategies.

## Introduction

Epigenetic and genetic alterations are two mechanisms in leukemia (1). Several factors, such as chromosomal translocations as well as genetic or epigenetic alterations, are involved in leukemogenesis (2,3). Abnormal methylation of DNA and histone modifications are important mechanisms in tumor suppressor silencing, contributing to leukemogenesis along with genetic alterations (1). The role of epigenetic alterations in the development of hematological malignancies has been identified in recent years (4,5). It was reported that many mechanisms leading to the gene activation or inactivation contribute to the tumor formation (6). On the other hand; drug resistance, including tyrosine kinase inhibitor resistance, has become a continuous clinical challenge; thus, the detection of abnormal genes specifically involved in leukemogenesis could be considered as prognostic biomarkers in disease classification serving as a new therapeutic protocol in leukemia (7,8). The purpose of this review was to introduce various genes inactivated in several leukemia types, and also evaluate their role in leukemia pathogenesis and prognosis. 

### Significance of inactivated genes in lymphoid leukemia

Inactivation of genes plays an important role in the pathogenesis and prognosis of lymphoid leukemia. Epigenetic mechanisms are the most prevalent inactivation ones in lymphoid leukemia and involve the genes implicated in several cellular mechanisms, including gene expression and transcription, cell- cycle regulation and apoptosis (Table 1) (9,10). 

**Table 1:** Inactivated genes in leukemia types


Gene	Chro.	Function	Mechanism of inactivation	Leukemia	Type of sample	Ref

*CDKN2A(p16^INK4a^)*	9p21	Tumor suppressor/G1-S cell-cycle control	Deletion	ALL	BM	(8, 11-13)
*MTAP*	9p21	Major role in polyamine metabolism	Deletion	ALL	BM	(14)
*CDKN2A(p14^ARF^)*	9p21	Cell-cycle control/Apoptosis regulation/Tumor suppressor	Deletion	ALL	BM	(14)
*P21^CIP1/WAF1/SDI1^*	6p21.2	Cyclin-dependent kinase inhibitor	Promotermethylation	ALL	BM	(3)
*BIM*	2q13	Pro-apoptotic BH3-only bcl2 family member/Tumor suppressor in B cell	Promoter methylation	ALL	BM	(15)
*Hsa-miR-124a*	8p23.1	Post-transcriptional regulation of gene expression	Promoter methylation	ALL	BM	(16)
*DKK-3*	11p15.1	Wnt antagonist/Tumorsuppressor	Promoter methylation	ALL	BM	(17)
*WIF1*	12q14.3	Wnt antagonist	Promotermethylation	ALL	BM	(18)
*ASPP1*	14q32-33	P53 costimulator	Promotermethylation	ALL	BM/HL-60, Jurkat, K-562 cell line	(19)
*EPHB4*	7q22	Receptor tyrosine kinase/Tumor suppressor	Promotermethylation	ALL	BM	(20)
*EFNB2*	13q33	Ephrin	Promotermethylation	ALL	BM	(20)
*EFNA5*	5q21	Ephrin	Promotermethylation	ALL	BM	(20)
*DBC1 / BRINP1*	9q33	Cell cycle arrest in G1/Tumor suppressor	Promotermethylation	ALL	BM	(21)
			Deletion		NALM-20/TOM-1 cell line	
*TES*	7q31.2	Tumor suppressor/Cell-matrix adhesions/Cell-cell contacts and to actin stress fibers	Promotermethylation	ALL	BM	(22)
*FHIT*	3p14.2	Histidine triad protein (HIT) family/Tumor suppressor	Promoter methylation	MLL	PB/BM	(23)
*SLC5A8*	12q23.1	Tumor suppressor/Transporter of endogenous monocarboxylates	Promoter methylation	MLL-PTD	PB/BM	(24)
*NOTCH3*	19p13.2-p13.1	Notch-Hes pathway	Promoter methylation	B-ALL	BM	(25)
*HES4*	1p36.33	Transcriptional repressor	Promoter methylation	B-ALL	BM	(25)
*HES5*	1p36.32	Transcriptional repressor	Promoter methylation/Histone deacetylation	B-ALL	BM	(25)
*BMP6*	6p24-p23	Regulators of cell proliferation, differentiation and apoptosis	Promoter methylation	ATL	PB/BM	(26)
*PTPN2*	18p11.3-p11.2	Cell growth/Negative regulator of the JAK-STAT pathway	Deletion	T-ALL	BM	(27)
*RIZ1*	1p36.21	Tumor suppressor/A member of a nuclear histone/Protein methyltransferase superfamily	Promoter methylation	T-ALL	BM	(28)
*CDKN2B/ P15^INK4B^*	9p21	G1-S cell-cycle control/Tumor suppressor	Deletion	ALL	BM	(8, 12-14, 29, 30)
			Promoter methylation	AML		
*NDRG2*	14q11.2	Tumor suppressor/Cellular stress	-	AML	HL60/U937/NB4/HT93 cell line	(31, 32)
			Deletion/Promoter methylation	ATLL	PB	
*CHD5*	1p36.31	Chromatin remodelingGene transcription	Promoter methylation	ALL/AM/CML	BM	(33)
*KLF2*	19p13.11	Zinc-finger transcription factors	-	T cell leukemia	Jurkat cell line	(34)
				AML	BM	
*SHP1*	12p13	JAK-STAT signaling pathway inhibitor/Tumor suppressor	Promoter methylation	ATL /AML/ ALL/CML	BM/PB	(35)
*IKZF1*	7p12.2	Transcription factor	Deletion/Mutation	B-ALL	PB/BM	(36)
				CML (blast crisis)		
*E-cadherin (CDH1)*	16q22.1	Maintenance of the epithelial phenotype/Mediated by a Ca11-dependent/Homotypic cell-cell adhesion	Promoter methylation	CLL/AML/ALL	PB/BM	(37)
*sFRP1*	8p12-11.1	Wnt antagonist	Promoter methylation	CML/ALL	BM	(38, 39)
				CLL	PB	
*ATM*	11q22-q23	Apoptosis/Cell cycle checkpoint	Mutation of the coding region	CLL	Tumor	(40)
*TP53*	17p13.1	Cell cycle arrest/Apoptosis	Mutation of the coding region	CLL	Tumor	(40)
*miR-15a*	13q14.2	Post-transcriptional regulation of gene expression	Histone deacetylation	CLL	-	(41)
*miR-16*	13q14	Post-transcriptional regulation of gene expression	Histone deacetylation	CLL	-	(41)
*miR-29b*	7q32.3, 1q32.2	Post-transcriptional regulation of gene expression	Unknown	CLL	-	(41)
*PTPRO*	12p13.3-p13.2; 12p13-p12	Receptor-type protein tyrosine phosphatases/Tumor suppressor	Promoter methylation	CLL	PB	(9)
*KLF4*	9q31.2	Zinc-finger transcription factors	Promoter methylation	CLL	PB	(42)
*APAF-1*	12q23	Initiates apoptosis	-	B-CLL	PB	(43)
*NUR77*	12q13	Tumor suppressor/Transcriptional activator	HDAC inhibition	AML	PB/BM	(44)
*NOR1*	9q22	Tumor suppressor/Transcriptional activator	HDAC inhibition	AML	PB/BM	(44)
*NDRG1*	8q24.3	Cellular stress/Cell growth/Differentiation	-	AML	HL60/U937/NB4/HT93 cell line	(32)
*KLF5*	13q22.1	Zinc-finger transcription factors	Promotermethylation	AML	BM	(34)
*FANCA*	16q24.3	Fanconi anemia, complementation group A	Deletions	AML	BM	(45)
*C/EBPδ*	8p11.2-p11.1	Transcription factor/Tumor suppressor	Promoter methylation	AML	PB	(46, 47)
*SOCS-1*	16p13.13	Suppressor of cytokine signaling/Tumor suppressor	Promoter methylation	AML	BM	(48)
*CAV1*	7q31.1	Major structural component of caveolae	-	AML	HL60 cell line	(49)
*NF1*	17q11.2	Tumor suppressor	Nf1 deﬁciency	AML	B106, B114 and B117 cell line	(50)
*IRF-4*	6p25-p23	Transcription factor	Promoter methylation	CML/ AML/ CMMoL	PB	(51)
* CDH13*	16q24	Cell recognition/Adhesion/Tumor suppressor	Promoter methylation	CML	PB	(52)
*SOCS-3*	17q25.3	Suppressor of cytokine signaling	Promoter methylation	CML	K562 cell line	(53)
*SARI/ BATF2*	11q13.1	Tumor suppressor	-	CML	PB	(54)
*PU.1*	11p11.2	Transcription factor	Unknown	CML	BM	(55)


ALL; Acute lymphoid leukemia, CLL; Chronic lymphoid leukemia, CML; Chronic myelogenous leukemia, AML; Acute myeloid leukemia, and BM; Bone marrow.

**Fig.1:**
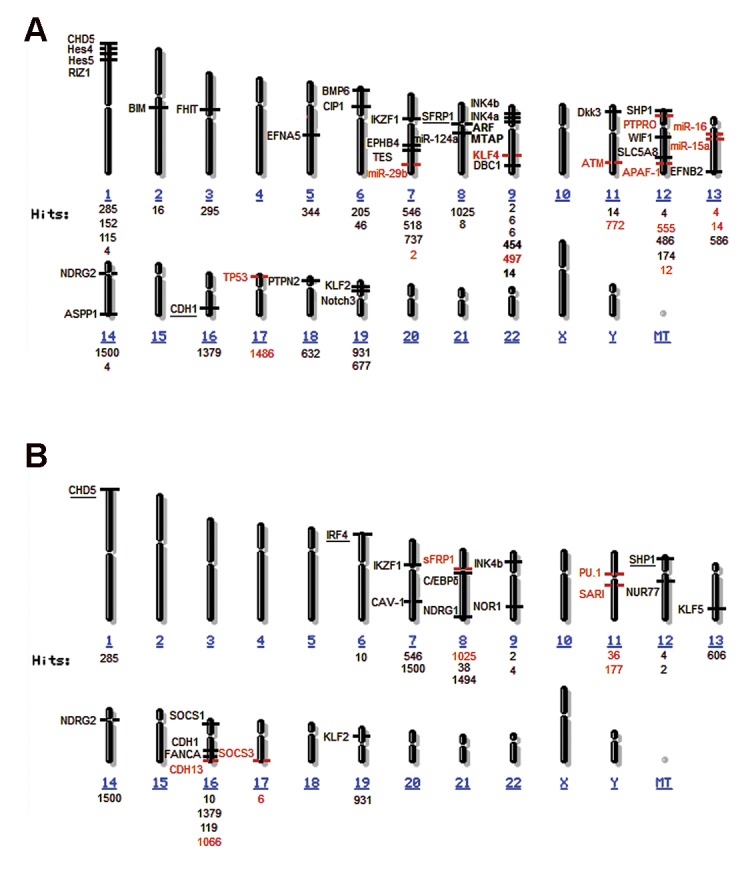
The maps of inactivated genes in leukemia. A. The maps of inactivated genes in lymphoid leukemia. Black genes; Inactivated genes in ALL, Red genes; Inactivated genes in CLL, Underlined genes are inactivated in both ALL and CLL and B. The maps of inactivated genes in myeloid leukemia. Black genes; Inactivated genes in AML, Red genes; Inactivated genes in CML, Underlined genes are inactivated in both AML and CML. ALL; Acute lymphoid leukemia, CLL; Chronic lymphoid leukemia, CML; Chronic myelogenous leukemia, and AML; Acute myeloid leukemia.

Method of obtaining map of genes. Genes of living organisms are usually represented by a long nucleotides molecule that makes up DNA. Bioinformatics is an integrated scientific discipline which addressing the use of computers to search or illustrate the information about genes. Here, we illustrate these principles using a new visual analytics tool named MapView (https://www.ncbi.nlm.nih.gov/mapview/) to facilitate the representation of a previously published set of gene data in human with leukemia (Fig.1) (56).

### Regulators of gene transcription

Human chromodomain helicase DNA binding protein 5 (CHD5), Krüppel-like factor 2 (KLF), Retinoblastoma protein-interacting zinc finger 1 (RIZ), and IKAROS family zinc finger 1 (IKZF1) are among the genes that regulates gene transcription, and are inactivated in acute lymphoid leukemia (ALL). CHD5, one of the nine members of the CHD family, is characterized by the unique combination of chromatin organizing modulator, helicase and DNA-binding domains (57). This gene acts as a chromatin remodeling protein. Expression of a tumor-suppressive network, including P16 and P19, encoding by cyclin-dependent kinase inhibitor 2A locus, facilitates suppression, while loss of CHD5 increases proliferation (58). The expression of this gene is generally reduced in human leukemia cell lines. CHD5 mRNA and protein expression are significantly lower in ALL patients in comparison with normal mononuclear cells (NMCs); thus, CHD5 can be used as a biomarker panel for hematopoietic malignancies even for therapeutic approaches (Figes.1A, 2) (33). KLF2, a member of KLF family of zinc-finger transcription factors, is another inactivated gene in ALL. KLF2 inhibits Jurkat leukemia cell growth via upregulation of cyclin-dependent kinase inhibitor. This factor has transactivation and inhibitory domains, both of which are involved in inhibition of cell proliferation; however, the transactivation domain is involved in the inhibition of DNA synthesis. P21WAF1/CIP1 induction is a KLF2 mechanism for cell cycle arrest and suppression of T-cell leukemia growth. This is a P53-independent induction and can be considered as a therapeutic target for leukemia since it is effective upon Jurkat leukemia with mutated P53 (Table 1) (59).

The reduced *RIZ1* expression is associated with leukemogenesis in adult ALL. RIZ1 is the protein encoded by *RIZ* gene, having a positive regulatory (PR) domain and transcriptional repression function. *RIZ1* promoter is methylated; thus its expression is reduced in T-ALL. *RIZ1* is a T-ALL specific tumor suppressor gene. Further studies are needed to elucidate the inactivation mode of *RIZ1* (28).

Deletion or mutation of IKAROS (*IKZF1*) is associated with minimal residual disease in *BCR-ABL1*-positive ALL, a poor outcome as well as high relapse rate in B-cell-progenitor ALL (60). IKAROS is a transcription factor playing an essential role in lymphopoiesis (61). *IKZF1* gene aberrations are associated with a poor outcome in B-ALL and have a high risk of relapse in leukemia (60). Deletion of *IKZF1* has been reported in 83.7% of BCR-ABL1-positive ALL cases. Aberrant RAG-mediated recombination is responsible for the deletions (36). In general, detection of *IKZF1* alterations upon diagnosis shows a high risk of treatment failure (60).

### Post-transcriptional regulators of gene expression

MicroRNAs (miRs) play an important role in the pathogenesis and prognosis of leukemia through post-transcriptional regulation. MiR-124a is a tumor suppressor involved in the pathogenesis of ALL. Epigenetic regulation of *hsa-miR124a* increases *CDK6* expression, leading to abnormal ALL cell proliferation both *in vitro* and *in vivo*. CDK6 is an oncogene playing a role in cell proliferation and differentiation. Hypermethylation of *hsa-miR-124a* is an independent prognostic factor for disease-free survival (DFS) as well as overall survival (OS) in ALL patients which is associated with a poor prognosis (16, 62).

Deletions in chromosome 13 [del (13q14)] are among the aberrations observed in chronic lymphoid leukemia (CLL) patients, result in decreased expression of *miR-15a* and *miR-16*. MCL-1 and BCL-2 are targets of miR-15a and miR-16. Low levels of *miR-15a* and *miR-16* in combination with selective loss of *miR-29b* may contribute to the pathobiology of CLL.*MiR-29b* is another miR, which is decreased in CLL.*MiR-29b* acts as a tumor suppressor targeting ,*Mcl-1, SP1, DNMT3a, DNMTb, Tcl-1* and *Cdk6* in CLL (41). The expression of this miR is reduced through an unknown mechanism in aggressive CLL and is associated with a poor prognosis (63).

### Cell cycle regulators

Deletions in cyclin-dependent kinase inhibitor 2A (*CDKN2A*) locus is a common mutation in T-ALL; so, *CDKN2A* tumor suppressor locus is disrupted in 90% of T-ALL cases (Table 1) (64, 65). *CDKN2A* is a tumor suppressor acting via INK4a/p16 and ARF/p14 proteins. This tumor suppressor functions upstream of retinoblastoma (Rb) gene to control the cell cycle arrest (65). Inactivating mutations in *CDKN2A* locus disrupt both Rb and P53 tumor suppressor pathways. In addition to *CDKN2A, CDKN2B/P15INK4B* is deleted in a significant fraction of ALL cases but it is always associated with *CDKN2A* deletion (Figes.1A, 2) (14).

Homozygous deletion of *P16, P14,* and *P15* is prognostic and affects the OS of adult B-ALL patients. However, methylation in the above-mentioned genes has no impact on survival of these patients (13). Moreover, *INK4* deletion is associated with prognosis in childhood ALL as an independent factor (11, 12), so any *P16* deletion is a major independent risk factor for relapse as well as a major independent negative prognostic indicator in pediatric ALL (11). Pediatric ALL with *INK4* deletion tends to relapse approximately one year later (median first-remission duration approximately 2.1 years versus approximately 3 years) but is not associated with event-free survival (pEFS) (12).

CDKI p21CIP1/WAF1/SDI1 is another CDKI. Hypermethylation of *P21* gene is a factor of poor prognosis in both childhood and adult ALL and patients with hypermethylation of *P21* show poorer DFS compared to those with normal methylation (3). Therefore, *INK4* deletion and *P21* methylation can have important clinical outcomes in ALL patients and will help in the selection of treatment and also be the basis for new therapeutic approaches.

Gene inactivation is not always associated with disease outcome in patients. DBC1 is involved in the pathogenesis of ALL despite the lack of a significant correlation between *DBC1* and relapse rate, mortality, DFS, and OS (21). DBC1 is located in the cytoplasm and leads to cell cycle arrest in G1 or at least slower G1 transition having an antiproliferative effect and which leads to apoptosis indirectly (66).

### Apoptotic genes

From among the genes involved in apoptosis, apoptosis-stimulating protein of P53 (*ASPP1*), bone morphogenetic protein (*BMP*) 6 and *BIM* are involved in the pathogenesis of ALL. Ataxia telangiectasia mutated (*ATM*), tumor protein P53 (TP53), and apoptosis protease-activating factor 1 (*APAF-1*) play a significant role in the pathogenesis of CLL (15, 19, 43, 67, 68).

ASPP family members, including ASPP1, ASPP2, and iASPP are effective upon P53 function. ASPP1 and ASPP2 activate P53 through induction of pro-apoptotic genes such as *BAX* and *PIG3* but iASPP acts as an activator of P53. ASPP1 methylation and inactivation is more frequent in adult ALL and T-ALL relative to childhood ALL and B-ALL, respectively; hypermethylation does not occur in the ASPP2 promoter (Figes.1A, 2) (69). Decreased *ASPP1* expression in leukemic cell lines is associated with increased *iASPP* expression. Therefore, alterations of *ASPP* play an important role in the pathogenesis of hematological neoplasms (69, 70). In addition, *ASPP1* can be considered as a factor of poor prognosis since the risk of relapse and mortality is higher in ALL patients with methylated *ASPP1* in comparison with those having unmethylated *ASPP1* (19).

BMP6 is a member of tumor growth factor (TGF)-β superfamily of multifunctional cytokines (71). This gene is highly methylated in ATL and to a lower extent in ALL and CLL. The degree of methylation is higher in aggressive types of ATL, and chronic ATL cases with BMP-6 promoter methylation are more aggressive clinically. The *BMP-6* promoter methylation may thus be a new biomarker to predict the progression to acute stages in chronic ATL patients however further research is needed in this field (26).

BIM is a pro-apoptotic factor that plays an important role in development and homeostasis of the lymphoid system and acts as a tumor suppressor in B-cells (72). In general, the balance between pro- and anti-apoptotic molecules is disrupted in many leukemic cells, leading to resistance to apoptosis, such that the imbalance between pro- and anti-apoptotic proteins of BCL-2 family results in the development of ALL and drug resistance (73, 74). The absence of *BIM* causes malignant B-cell resistance to glucocorticoid *BIM* (72). Thus *BIM* expression is lower in high-risk childhood ALL and is associated with slow early response to a standard 4-drug combination (15).

**Fig 2:**
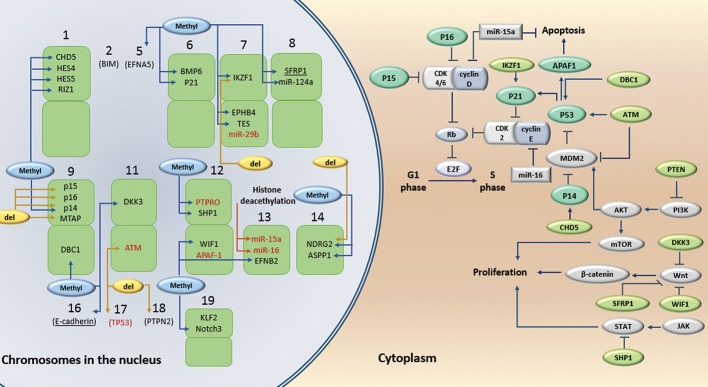
Inactivated genes in lymphoid leukemia. Black genes; Inactivated genes in ALL, Red genes; Inactivated genes in CLL, Underlined genes are inactivated in both ALL and CLL. APAF; Apoptosis protease-activating factor 1, ATM; Ataxia telangiectasia mutated, BMP6; Bone morphogenetic protein-6, CDK; Cyclin-dependent kinase, CHD5; Chromodomain helicase DNA binding protein 5, Dkk3; Dickkopf, EFN; Ephrin, IKZF1; IKAROS family zinc finger 1, JAK; Janus kinase, KLF; Krüppel-like factor, MDM2; Mouse double minute 2 homolog, miR; Micro RNA, MTAP; S-methyl-5'-thioadenosine phosphorylase, mTOR; Mammalian target of rapamycin, NDRG; N-myc downstream regulated gene, PTEN; Phosphatase and tensin homolog, PTPN2; Protein tyrosine phosphatase non-receptor type 2, PI3K; Phosphoinositide 3-kinase, SFRP1; Secreted frizzled-related protein 1, SHP; SH2-containing phosphates, STAT; Signal transducers and activators of transcription, TP53; Tumor protein P53, and WIF1; WNT inhibitory factor 1.

Aberrant *P53* activation is associated with poor prognosis in CLL patients. This disorder may occur directly as a result of *TP53* gene mutation or indirectly via *ATM* inactivation (40). ATM is the central component of signal transduction pathway (75). CLL patients with complete loss of ATM function have a poor response to cytotoxic chemotherapeutics in vitro due to biallelic *ATM* defects and are associated with poorer clinical outcome (67). Respectively Dysfunctional mutation in *TP53* and *ATM* accounts for 80% of 17p- and 36% of 11q- cases (76, 77). These mutations are associated with poor responses to purine analogue-containing chemoimmunotherapy and shorter survival (67). Although a loss of *APAF1* alone is not effective upon disease prognosis, it has prognostic relevance in the small subset of P53-mutated B-CLL patients. APAF-1 is the transcriptional target of P53 and plays a role in linking the mitochondrial apoptotic pathway to caspase cascade (Fig.2) (43).

### Wnt pathway antagonists

Wnt signaling plays a pivotal role in the proliferation of thymocytes and pro-B cells. Also Wnt proteins are thus growth factors for progenitor cells of both B- and T-cell lineages (78). Wnt signals are essential for survival and growth of lymphocyte progenitors. Furthermore; impaired Wnt signaling can be a mechanism of lymphoid leukemogenesis (Fig.2) (18). Dickkopf (*DKK3*) gene, negatively modulating Wnt7A signaling, is highly silenced in ALL (17). Generally, silencing of Wnt antagonists (*DKK3, WIF1, sFRPs* and *DACT1*) by promoter methylation leads to activation of canonical Wnt/β- catenin signaling pathway in ALL, which plays a role in the pathogenesis of the disease (18). If silencing of *DKK-3* expression occurs in early stages of ALL pathogenesis, it plays an important role in disease outcome. *DKK-3* methylation and silencing are an independent prognostic factor in predicting DFS in ALL (17). Overally, hypermethylation and silencing of Wnt inhibitors in ALL is associated with poor prognosis (18). Hypermethylation of *sFRP1* has also been reported in CLL patients (79).

### Suppressors of JAK/STAT pathway

SH2-containing phosphatase (*SHP1*) is a non-receptor protein tyrosine phosphatase (PTP) expressed at high levels in hematopoietic cells. SHP1 inhibits growth-promoting signaling such as Janus kinase/signal transducers and activators of transcription (JAK/STAT) (Fig.2). Hypermethylation and silencing of *SHP1* gene are observed in a wide range of hematopoietic malignancies (35, 80). SHP1 gene is basically methylated in blast crisis of adult T-cell leukemia-lymphoma (ATLL) from carrier status to acute or lymphoma type ATLL as well as during progression to aggressive ATLL (81). Therefore, evaluation of SHP1 as a prognosis factor in ATLL is recommended.

### Notch-HES Pathway

Notch receptor signaling pathway is involved in many cellular functions such as hematopoietic stem cell self-renewal, cell lineage commitment, maturation and differentiation (82, 83). Also Notch signaling regulates T- and B-cell lineage commitment (84). It increases T-cell proliferation in neoplastic transformation of T lymphoid progenitors which results in malignancy (85). Overexpression of the active forms of Notch receptors (*ICN1-4*) or Notch downstream target gene *hairy and enhancer of split-1* (*HES1*) in human B-cell leukemia/lymphoma can lead to apoptosis (86). Notch pathway genes *Notch3* and *HES5* are hypermethylated in human B-ALL cases but the molecular mechanisms of oncogenic and tumor suppressive activity of Notch are not well known (25).

### Regulators of PI3K/Akt pathway

N-MYC downstream regulated gene (NDRG)-2 is a PTEN-binding protein recruiting protein phosphatase 2A (PP2A) to PTEN. NDRG2 interacts with PTEN and activates its phosphorylation (31). Genetic and epigenetic inactivation of *NDRG2* in ATLL cells increases PTEN phosphorylation and reduces its activity, following by increased activity of phosphoinositide 3-kinase (PI3K)-AKT pathway and enhanced proliferation (Fig.2) (31, 87). Increased activation of PI3K-AKT plays an important role in the development of leukemia (87). Therefore, *NDRG2* can be considered as a prognostic factor in future studies.

### Cell adhesion

Expression of E-cadherin (Cadherin1:*CDH1*) gene, which is commonly methylated in ALL and CLL leukemia cells, is not detectable in lymphoid blasts (37, 88). CDH1 is involved in homotypic cell-cell adhesion. Inhibition of Wnt pathway is another function of CDH1. Lack of *E-cadherin* expression is one reason for increased activity of Wnt signaling in CLL cells (37). TESTIN (TES) is a component of focal adhesion complex involved in cell-matrix adhesions and cell-cell contacts. Silencing of TES may contribute to ALL pathogenesis through adhesion and interference with normal interactions between progenitors and stroma since it increases the mobility of immature progenitors, leading to premature release from bone marrow (BM) niches. *TES* gene expression is decreased to a higher extent in B-ALL relative to MLL-translocation ALL and T-ALL (22). Hence, it can be used to distinguish between lymphoid leukemia types.

### Significance of inactivated genes in myeloid leukemia

Genetic defects and also hypermethylation, can contribute to initiation and maintenance of AML (89). Hypermethylation of tumor suppressor genes is a commonly deregulated mechanism in acute myeloid leukemia (AML) and chronic myelogenous leukemia (CML) (54, 90). *CAV-1, NUR77, NOR1, P15INK4B* as well as the suppressor of activator protein-1 regulated by interferon *(SARI), SHP1* and *CDH13*, are respectively among these tumor suppressors in AML and CML (Figes.1B, 3) (30, 35, 44, 49, 52, 54, 80).

### Regulators of gene transcription

KLF5, CCAAT/enhancer binding protein (C/EBP) δ, NUR77, NOR1, PU.1, IKZF1, Interferon regulatory factor (IRF) 4 and CHD5 are among regulators of gene transcription in myeloid leukemia (33, 34, 44, 46, 51, 55, 60). KLF5 regulates the genes involved in regulation of cell growth, apoptosis, migration, and differentiation. The expression of this factor which is particularly increased in granulocyte lineagewhich plays a special role in granulocytic development (90, 91). Decreased *KLF5* expression causes reduced granulocytic differentiation in response to granulocyte-colony stimulating factor (G-CSF) signaling which is an essential factor for differentiation of APL cells in response to all-trans retinoic acid (ATRA) (90). The transcriptional target of KLF5 is cell cycle inhibitor of P15^INK4b^, which is usually inactive in AML because of promoter hypermethylation (92). The function of oncogenic fusion proteins like PML-RARα may result in decreased *KLF5* expression since these fusion proteins directly reduce the expression of tumor suppressors such as *P21 (CDKN1A)* (93). Hypermethylation detection at *KLF5* locus can help identifying the appropriate patients for specific therapy because demethylating agents such as 5-aza- 2-deoxycytidine (Decitabine), reactivating *KLF5* expression, have been successful in some clinical trials in AML (Table 1) (90, 94). Among C/EBP transcription factors expressed during the development of myeloid lineage, *C/EBPδ* is extensively silenced in AML. Although promoter of *C/EBPδ* is the major methylated gene in AML, there is no correlation between disease stage and its methylation, leading to silencing (65).

*NUR77 (NR4A1)* and NOR1 (*NR4A3*) are transcription factors involved in different cellular and physiological functions, including apoptosis, mitosis, inflammation, and differentiation (95- 98). Also *NUR77* regulates the induction of FAS-L, TRAIL, and pro-opiomelanocortin in lymphocytes (99). NUR 77 and Nor1 transcripts are decreasing significantly in leukemic blasts of AML patients in comparison with normal BM cells (44). Therefore, silencing of *NUR77* and *NOR1* plays an important role in the pathogenesis of AML. Intensive silencing of *NUR77* and *Nor1* occur not only in bulk leukemia cells but also in leukemia stem cells (LSCs) (Figes.1B, 3) (99). PU.1 is another transcription factor involved in myeloid development, controlling the expression of genes important for fate determination of both myeloid and lymphoid lineages. The role of *PU.1* in leukemic processes is related to its expression level, associating with AML if decreased. The PU.1 level is reduced in CML patients upon diagnosis but it will be increased after treatment with interferon-α or imatinib and return to normal hematopoiesis. Thus, it may be used as a factor to determine response to treatment (55). IRF4 belongs to IRF family with an important role in the regulation of several genes, including IFNs, interleukins, MHC class I/II, apoptosis, and differentiation/maturation (51). IRF4 is also a transcriptional regulator using as a useful marker in monitoring but not the screening of response to IFN-alpha in CML. The expression of this gene is decreased in CML, AML, and chronic myelomonocytic leukemia (CMMoL) patients, and increased expression of *IRF4* is associated with good response to IFN-alpha therapy (51).

### Regulators of the cell cycle

**Fig.3:**
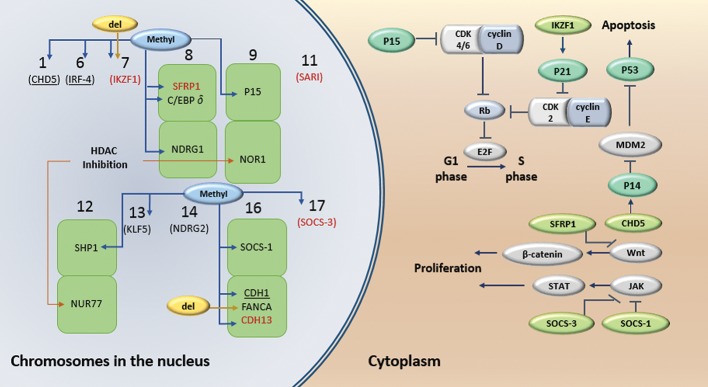
Inactivated genes in myeloid leukemia. Black genes; Inactivated genes in AML, Red genes; Inactivated genes in CML, underlined genes are inactivated in both AML and CML. CDH; Cadherin, CDK; Cyclin-dependent kinase, C/EBPδ; CCAAT/enhancer binding protein, delta, CHD5; Chromodomain helicase DNA binding protein 5, FANCA; Fanconi anemia, complementation group A, IKZF1; IKAROS family zinc finger 1, IRF-4; Interferon regulatory factor 4, JAK; Janus kinase, KLF; Krüppel-like factor, MDM2; Mouse double minute 2 homolog, NDRG; N-myc downstream regulated gene, SFRP1; Secreted frizzled-related protein 1, SOCS; Suppressors of cytokine signalling, SHP; SH2-containing phosphates, STAT; Signal transducers and activators of transcription, and TP53; Tumor protein P53.

P15INK4b, decreased in APL patients, inhibits cyclin D-CDK4/6 and results in cell cycle arrest in G1 (Fig.3). In addition, patients with methylated *P15* have a higher relapse risk and lower DSF, which is suggestive of poor prognosis (52). CAV- 1, the major structural component of caveolae, is a protein that plays an essential role in tumorigenesis. The *CAV-1* expression is reduced in HL-60 cell line. In fact, overexpression of *CAV-1* inhibits HL- 60 cell proliferation, induces apoptosis, arrests the cell cycle in G1 phase, and inhibits the activation of PI3K/AKT/mTOR signaling pathway (49).

### Cellular stress

NDRG1 is a protein induced as a result of cellular stress. The *NDRG1/2* expression increases during cell differentiation. *NDRG1* increases neutrophil differentiation via increasing the expression of key transcription factors of myeloid series, including *C/EBPδ* and *PU.1*. Decreased expression of *NDRG1* is associated with reduced cell differentiation in NB4 cells. In general, the *NDRG1/2* expression is reduced in primary AML in which the cells are blocked in early myeloid differentiation stages (32).

### Suppressors of JAK/STAT pathway

Suppressors of cytokine signaling (SOCS 1 and 3) are among the genes inactivated in myeloid leukemia. SOCS is a negative regulator of JAK/STAT signaling. This signaling regulates biological activities of the cell, including growth and differentiation (Fig.3) (53). SOCS1 is the most potent inhibitor of JAK in SOCS family acting as a tumor suppressor. SOCS1 is inactivated in AML patients because of promoter methylation (48). SOCS-3, which specifically targets STAT3, is inactivated in K562-R cells, is resulting in activation of STAT3 signaling and resistance mechanisms. Hypermethylation of SOCS3 may cause resistance to tyrosine kinase inhibitors in break point cluster-Abelson (BCR-ABL) positive CML because overactivity of STAT3 leads to unchecked cell proliferation (53). SHP1, one of the inhibitor of JAK/ STAT pathway, is subject to decreased expression in advanced-phase CML patients relative to the chronic phase. Therefore, the loss of SHP-1 function may play a key role in progression to blast crisis in CML (100).

### Cell adhesion

CDH13, a member of cadherin family involved in cell adhesion, is subject to decreased expression in CML patients. *CDH13* methylation is also associated with shorter median progression-free survival time in CML patients and predicts poor cytogenetic response to interferon α treatment (52).

## Discussion

Predictive modeling is a powerful implement to test a hypothesis, confirm an experiment, and also mimic a dynamics of complex system (101). Along with clear mechanistic understanding of dynamical systems, predictive models perform the simulation of a complex system in a predictive manner and relatively fast-time with no enormous costs of laboratory experiments. Especially in oncology, predictive models can be established by using available clinical or experimental data (102,104), as well as tumor progression and potential treatment options that can be assessed prior to clinical intervention (105,109). Walter et al. (110) assessed the effect of genetic profiling on prediction of therapeutic resistance and survival in adult acute myeloid leukemia and showed that genetic profiling rises the accuracy of multivariable models predicting therapeutic resistance in adults with newly diagnosed AML. Bou Samra et al. (111) built a 20-gene expression (GE)-based risk score that used to predictive overall survival and improving risk classification of patients with CLL. Also it showed that such predictive model represents a powerful tool for risk stratification and outcome prediction, which could be used to guide clinical and therapeutic decisions prospectively. Inactivated genes are involved in different cellular functions such as cell cycle, apoptosis and particularly gene transcription (Table 1) (15,18,19). Inactivation of *RIZ1, BMP6,* and *SHP1* is specific for T-ALL and *IKZF1* inactivation is for B-ALL. TES methylation is more pronounced in B-ALL relative to MLL and T-ALL while silencing of *FHIT* is a feature of MLL (23,26,28,35). Therefore, the mentioned genes can be used as additional diagnostic tests to differentiate leukemia types. 

Inactivation of genes is involved in leukemia prognosis that suggests poor prognosis of leukemia (Table 2). Inactivation of *BMP6* and *SHP1* from among the genes playing a role in ALL prognoses predicting the aggressive type of T-ALL (26,28). Inactivation of *miR-124a*, Wnt inhibitors and P21 has been associated with poor prognosis of ALL (3,16,18,62). In addition, inactive *IKZF1* and *ASPP1* are also associated with increased risk of relapse. *CDKN2A* and *CDKN2B* inactivation are associated with poorer OS of adult B- ALL but not with pEFS of childhood ALL (19,60). In CLL patients, inactivation of *miR-29b* and *APAF-1* (only in P53-mutated group) is a sign of poor prognosis (41,43). SHP-1 methylation is associated with progression to blast crisis, and *CHD13* inactivation is associated with shorter median progression- free survival time in CML patients (35,52). In AML patients, inactive *P15* is indicative of poor prognosis (30). 

**Table 2:** The effect of inactivated genes on prognosis of the leukemia


Inactivated gene	Effect on prognosis of leukemia	Leukemia	Reference

*IKZF1*	High risk of relapse in leukemia High risk of treatment failure	ALL	(60)
*miR-124a*	An independent prognostic factor for DFS and OS Is associated with a poor prognosis	ALL	(16, 62)
Wnt inhibitors^*^	Is associated with poor prognosis	ALL	(18)
*CDKN2A*	Is associated with poorer OS of adult B- ALLHigh risk of relapse in leukemiaNo correlation with pEFS of childhood ALL	ALL	(8, 11-13)
*CDKN2B*	Is associated with poorer OS of adult B- ALL High risk of relapse in leukemiaNo correlation with pEFS of childhood ALL	ALL	(8, 11-13)
*DBC1*	No correlation with relapse rate, mortality, DFS and OS	ALL	(21)
*ASPP1*	Is associated with a high risk of relapse and mortality	ALL	(19)
*P21*	Is associated with a poor prognosis Is associated with poorer DFS	ALL	(3)
*BMP-6*	May thus be a new biomarker to predict the progression to acute stages	Chronic ATL	(26)
*miR-29b*	Is associated with a poor prognosis	CLL	(63)
*APAF-1 and P53*	A predictor of poor prognosis	B-CLL	(43)
*C/EBPδ*	No correlation with disease stage	AML	(46)
*P15*	Higher relapse risk and lower DFSPoor prognosis	APL	(30)
*SHP-1*	It may play a key role in progression to blast crisis	CML	(100)
*CDH13*	Is associated with shorter median progression-free survival time	CML	(52)


ALL; Acute lymphoid leukemia, AML; Acute myeloid leukemia, ATM; Ataxia telangiectasia mutated, CLL; Chronic lymphoid leukemia, CML; Chronic myelogenous leukemia, IRF; Interferon regulatory factor, KLF; Kruppel like factors, SHP; SH2-containing phosphates, and SOCS; Suppressor of cytokine signaling proteins.

Gene inactivation is not always prognostic so the inactivation of *C/EBPδ* in AML, as well as *DBC1* inactivation, has no effect upon patient’s outcome (46,112). Prediction of response to treatment and screening for specific treatments is another important aspect of the inactivated genes (Table 3). Decreased *KLF5* can be used as a marker to identify AML patients for specific treatment with 5-aza-2-deoxycytidine (34). The consequence of neurofibromin 1 (*NF1*) gene inactivation in AML that confers Cytarabine (Ara-C) resistance through MAPK and mTOR pathways was reported formerly (50). In CML patients, *CHD13* reduction predicts poor response to IFNα, and also increased expression of *IRF-4* predicts a good response to this treatment (51,52). In these patients, increased expression of *PU.1* indicates a good response to treatment with interferon-α or imatinib and return to normal hematopoiesis (55). These genes can also be used as a complementary test to predict patients’ response to treatment. All or most of the papers that was mentioned to explain the inactivation of some genes in leukemia are original ones where primary Leukemia specimens were obtained by either freshly purified blood or frozen purified blast cells derived from leukemia patients at the moment of the Leukemia diagnosis. Therefore, it is conceivable that the inactivation/activation state of various genes could represent a clinical feature to take into account at least for the disease classification. 

**Table 3:** The effect of inactivated genes on treatment of leukemia


Gene	The effect of inactivated genes on treatment	Leukemia	Reference

*BIM*	Slow early response to standard 4-drug combination	Childhood ALL	(15)
*TP53 and ATM*	Poor responses to purine analogue	CLL	(43, 67)
*KLF5*	Reduced granulocytic differentiation in response to granulocyte-colony stimulating factor (G-CSF)	AML	(90)
*NF1*	Leading to Ara-C resistance	AML	(50)
*PU.1*	The expression of *PU.1* is increased after treatment with interferon-α or imatinib and return to normal hematopoiesis.	CML	(55)
*IRF4*	Increased expression of *IRF4* is associated with good response to IFN-alpha therapy	CML	(51)
*SOCS3*	May cause resistance to tyrosine kinase inhibitors	CML (Ph+)	(53)
*CDH13*	Predicts poor cytogenetic response to IFN-alpha treatment	CML	(52)


ALL; Acute lymphoid leukemia, AML; Acute myeloid leukemia, ATM; Ataxia telangiectasia mutated, CLL; Chronic lymphoid leukemia, CML; Chronic myelogenous leukemia, IRF; Interferon regulatory factor, KLF; Kruppel like factors, SHP; SH2-containing phosphates, and SOCS; Suppressor of cytokine signaling proteins.

## Conclusion

Inactivation of genes, which is mainly mediated by hypermethylation of gene promoters, plays an important role in pathogenesis and prognosis of leukemia. Generally, this review showed some genes inactivated only in leukemia (with differences between B-ALL, T-ALL, CLL, AML and CML). These differences could be considered as an additional tool to better categorize leukemia types. Furthermore, a diverse classification of Leukemias based on inactivated genes could represent complementary diagnostic tests to differentiate leukemia types, determine prognosis and a powerful method to address a targeted therapy of the patients, in order to minimize side effects of conventional therapies and to enhance new drug strategies. 
